# A Facile Green Synthetic Route for the Preparation of Highly Active γ-Al_2_O_3_ from Aluminum Foil Waste

**DOI:** 10.1038/s41598-017-03839-x

**Published:** 2017-06-15

**Authors:** Ahmed I. Osman, Jehad K. Abu-Dahrieh, Mathew McLaren, Fathima Laffir, Peter Nockemann, David Rooney

**Affiliations:** 1 School of Chemistry and Chemical Engineering, Queen’s University, David Keir Building, Stranmillis Road, Belfast, BT9 5AG Northern Ireland United Kingdom; 2 Chemistry Department, Faculty of Science - Qena, South Valley University, Qena, 83523 Egypt; 3 Centre for Nanostructured Media, School of Mathematics and Physics, Queen’s University Belfast, Belfast, BT7 1NN UK; 4 Department of Chemical and Environmental Sciences, Materials and Surface Science Institute, University of Limerick, Limerick, Ireland

## Abstract

A novel green preparation route to prepare nano-mesoporous γ-Al_2_O_3_ from AlCl_3_.6H_2_O derived from aluminum foil waste and designated as ACFL550 is demonstrated, which showed higher surface area, larger pore volume, stronger acidity and higher surface area compared to γ-Al_2_O_3_ that is produced from the commercial AlCl_3_ precursor, AC550. The produced crystalline AlCl_3_.6H_2_O and Al(NO_3_)_3_.9H_2_O in the first stage of the preparation method were characterized by single-crystal XRD, giving two crystal structures, a trigonal (R-3c) and monoclinic (P2_1_/c) structure, respectively. EDX analysis showed that ACFL550 had half the chlorine content (Cl%) relative to AC550, which makes ACFL550 a promising catalyst in acid-catalysed reactions. Pure and modified ACFL550 and AC550 were applied in acid-catalysed reactions, the dehydration of methanol to dimethyl ether and the total methane oxidation reactions, respectively. It was found that ACFL550 showed higher catalytic activity than AC550. This work opens doors for the preparation of highly active and well-structured nano-mesoporous alumina catalysts/supports from aluminum foil waste and demonstrates its application in acid-catalysed reactions.

## Introduction

Aluminum (Al) manufacture and expenditure are considered to be two sides of a coin and both are increasing. Aluminum is mainly produced from bauxite mines worldwide, reaching 270 million metric tonnes (Mt) in 2015 compared to 183 Mt in 2006^1^. Conversely, the Al consumption approximately tripled from 2006 to 2015 by 45 to 120 Mt, respectively, with a growth rate of 4% per year. To meet the dramatic increase in the Al consumption, a large amount of bauxite production is needed, which generates significant levels of waste such as tailings, red mud, emissions of perfluorocarbon and CO_2_ gases during the production process. Recycling this Al waste is crucial for the environment; in fact, recycling 1 kg of Al saves 8 kg of bauxite, 4 kg of chemical products and 14 kW of electricity^2^. Al waste can be recycled to produce various useful products. Li *et al*.^3^ successfully prepared γ-Al_2_O_3_ from oil shale ash waste which consists of 10.66 wt% Al_2_O_3_; however, they used a high calcination temperature at 700–800 °C to remove residual organic compounds and expensive chemicals such as Eu(NO_3_)_3_, urea and surfactant. Chotisuwan *et al*.^4^ managed to prepare γ-Al_2_O_3_ at low calcination temperature (500 °C) from Al cans, however, they used a costly preparation method by using isopropanol and mercury iodide. Al foil is one of the biggest Al waste sources which is difficult to recycle; thus the end-of-life options are either landfilling or incineration^5^. The global Al foil market had a size of 3.4 Mt in 2010. In the UK alone in 2001, 160,000 t of Al waste were sent to landfill^6^. Converting the Al foil into useful products is an environmental issue; few efforts were performed in previous publications^7^. Recently, Al foil was used in H_2_ production by converting it into active Al powder that reacts with water producing H_2_^7^ or by using Ca(OH)_2_ to remove the surface oxide layer and initiate the hydration reaction of Al foil^8^. Furthermore, α-Al_2_O_3_ was prepared at a low calcination temperature (1100 °C) for the industrial applications such as refractory materials, however, aqua regia was used during the preparation procedures, consequently, the release of NO_x_ emissions is possible during the preparation, which is harmful to the environment^9^. Qin *et al*.^10^ demonstrated a facile and a robust method to prepare ordered anodic aluminum oxides with continuously tunable inter-pore distances. Ye *et al*.^11^ studied the catalytic decomposition of formaldehyde using Flexible Mg-Al layered double hydroxide supported Pt on Al foil. No research has been performed on the potential preparation of ultra-pure single-crystalline AlCl_3_.6H_2_O and Al(NO_3_)_3_.9H_2_O subsequently highly active γ-Al_2_O_3_ from Al foil and its applications in acid-catalysed reactions as a catalyst/support. Converting Al foil into a very fine alumina catalyst/support is of importance for both academia and industry application (e.g. polymerization, reforming, dehydration and hydrogenation^12^). Aluminum oxide, alumina (Al_2_O_3_), is one of the most attractive ceramic materials for its various applications due to its thermal, chemical and mechanical stability^13, 14^.

Nonporous crystalline γ-Al_2_O_3_ offers good surface characteristics in heterogeneous catalysis, such as large specific surface area, pore size, pore volume and a highly active site concentration on its surface. The unique properties of mesoporous nanoparticles make it of interest for applications in industrial catalysis such as the dehydration of methanol to DME and the total methane oxidation. The preparation of mesoporous structures in heterogeneous catalysis is essential. Tang *et al*.^15^ prepared robust highly active electro-catalytic materials with large mesoporous (up to 16 nm). Well-defined morphology and consistency in particle sizes during the preparation of mesoporous materials are crucial with a broad range of applications^16^. Furthermore, controlling the meso-structure, particle sizes, morphology along with the mechanism of formation are of great interest in catalysis^17^.

Yaripour *et al*.^18^ studied the effect of modification of γ-Al_2_O_3_ with (0–15%) SiO_2_ and reported the % methanol conversion of 64.9% for the unmodified γ-Al_2_O_3_ at a reaction temperature of 300 °C and LHSV = 15.6 mL/(h g_cat_). Osman *et al*.^19^ considered the effect of the precursor on the performance of alumina in the methanol dehydration reaction and reported 51% methanol conversion for the commercial γ-Al_2_O_3_ at a reaction temperature of 300 °C with a WHSV of 12.1 h^−1^. In Alamolhoda *et al*. work^20^, nano γ-Al_2_O_3_ catalyst was synthesized through precipitation process for methanol dehydration reaction in a slurry batch reactor and reported 80% methanol conversion at 325 °C. Hosseini *et al*.^21^ studied the effects of different precipitating agents on the performance of γ-Al_2_O_3_ nanocatalysts, where the results showed that the catalyst prepared by ammonium carbonate showed the highest yield of DME and the selectivity toward the DME was 100% in the temperature range of 250–375 °C with 70% methanol conversion at reaction temperature of 300 °C with WHSV of 20 h^−1^. Kim *et al*.^22^ reported 50% methanol conversion over alumina catalyst at a reaction temperature of 305 °C with SV of 10 h^−1^.

The above discussion leads to the conclusion that converting Al foil into mesoporous γ-Al_2_O_3_ using a cost-effective green synthetic route is highly desirable. Herein, we demonstrate a novel eco-friendly synthesis of mesoporous γ-Al_2_O_3_ from Al foil (ACFL550) with surface and bulk characteristics better than commercial γ-Al_2_O_3_ (AC550). A comparison between the catalytic activity of the pure and modified (ACFL550 and AC550) showed that ACFL550 catalysts were more active than those of AC550. These results open doors for the preparation of highly active and well-structured alumina catalysts and supports from aluminum foil waste and demonstrate its application in acid catalysed reactions.

## Results and Discussion

**Figure 1:**
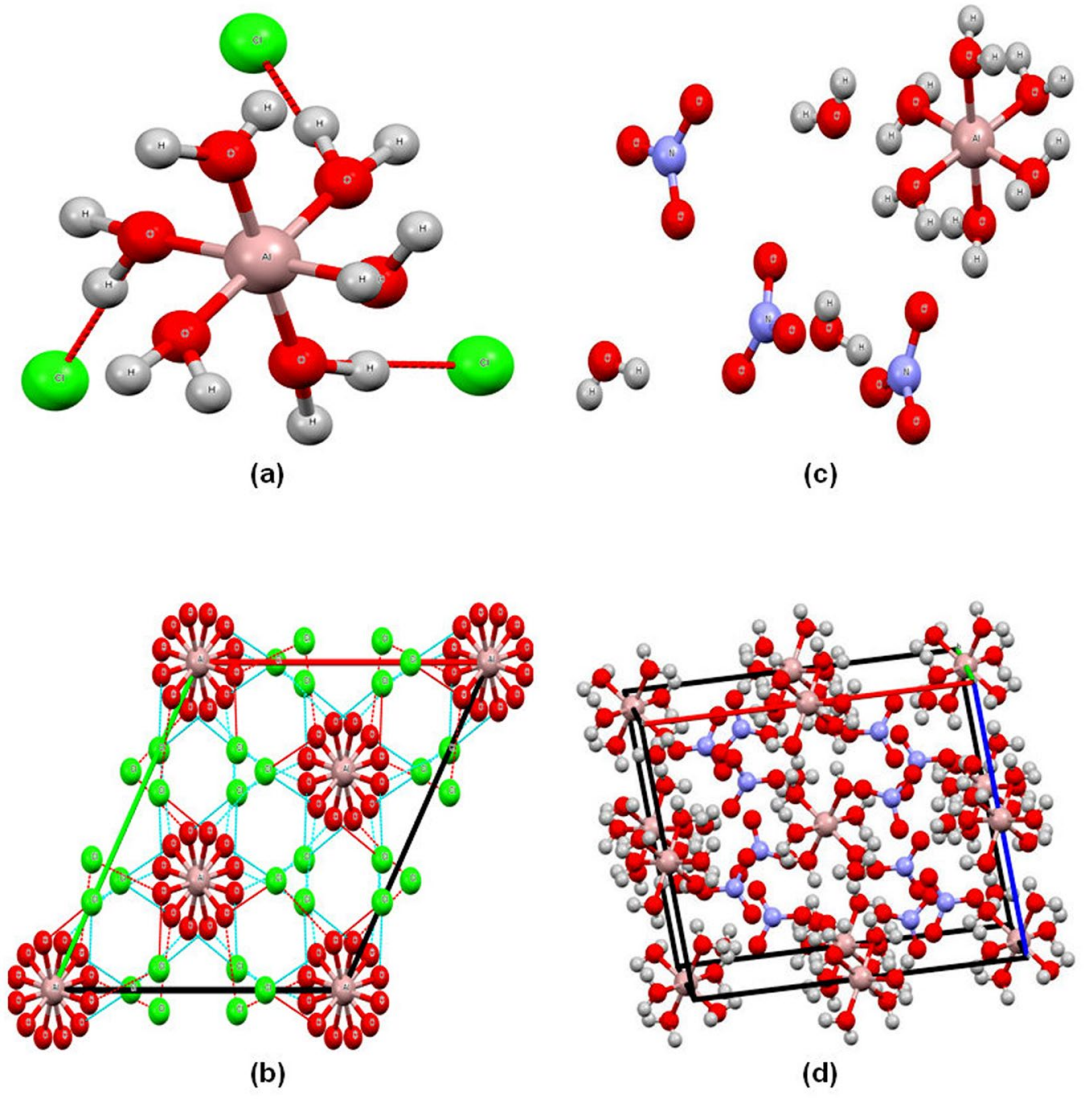
X-ray single crystal of homemade aluminium chloride hexahydrate in (**a** and **b**) and aluminium nitrate nonahydrate in (**c** and **d**)derived from aluminum foil waste, where images (**b** and **d**) depict the packing cubic crystal structure viewed along [001].

Figure 1(a) shows the X-ray single crystal structure of the AlCl_3_.6H_2_O produced by the experimental procedure described in the methods. The structure consists of chains of Al(H_2_O)_6_^3+^ units and hydrogen bonded chloride anions bridging between the chains to result in a network structure^23^. The bond lengths of Al^3+^-O are 1.881(2) Å and this is in agreement with work by Buchanan and Harris who reported 1.88(2) Å^23^. The bond angle of Al-O-Al is 90.04(11)°, while it was earlier reported at 90(1)°, indicating octahedral coordination^23^. Each water molecule and the two chlorine anions are linked together by hydrogen bonding as shown in Figure S1(b), resulting in the structure [Al(H_2_O)_6_]Cl_3_. The packing in the unit cell is shown along the [001] in Figure 1(b) (the supplementary video 1). The crystal structure of trigonal AlCl_3_.6H_2_O is matching the one described by Buchanan and Harris for Al(H_2_O)_6_^3+^-3Cl^−^^23^. The comparison between the lattice parameters and space group of our work and that done by Buchanan and Harris showed identical results (Table S1). However, the re-determination of the structure in our work showed better results due to the utilization of a modern instrument.

Figure 1(c) shows the X-ray single crystal structure for the Al(NO_3_)_3_.9H_2_O produced by the experimental procedure described in the methods. The structure consists of Al(H_2_O)_6_, 3(NO_3_), 3(H_2_O), and the asymmetric unit contains Al (H_2_O)_6_^3+^ (Al^3+^ is coordinated with 6 H_2_O molecules), 3(NO_3_)^3−^ and 3 H_2_O molecules which are not coordinated to the Al^3+^ ions. It is apparent that there are three independent water molecules, which are not coordinated with Al^3+^ ion; two of them are situated close to the Al (H_2_O)_6_^3+^ layer and participate in the bonding between octahedral in the same layer, while the third water molecule is situated between the layers and links together 3 nitrate ions and 2 H_2_O molecules. The bond lengths of Al^3+^-O lie in the range of 1.868–1.896 Å and in agreement with the work by Hermansson who reported 1.868–1.897 Å^24^. These ions linked together by hydrogen bonding as shown in Figure S1(c), giving the structure of [Al(H_2_O)_6_](NO_3_)_3_.3H_2_O (the supplementary video 2). The observed systematic extinction of reflections is in agreement with space group p2_1_/c which is for the monoclinic cubic structure as shown in Figure 1(d). The crystal structure of monoclinic Al(NO_3_)_3_.9H_2_O is similar to that described by Hermansson for Al(NO_3_)_3_.9D_2_O^24^. The comparison between the lattice parameters and space group of our work and that done by Hermansson showed the same results (Table S2). It is not surprising that the crystal lattice volume in Hermansson work was bigger than that of our work which showed 1452.3 (2) and 1418.29(3) Å^3^ at analysis temperature 295 K (21.85 °C) and 123.9 (−149.25 °C), respectively. This is due to the difference in the temperature during the analysis; lower temperature shrinks the crystal structure and consequently led to small crystal lattice volume. It is worth noting the similarities and differences between the lattice parameters and space group for the homemade single crystals of AlCl_3_.6H_2_O and Al(NO_3_)_3_.9H_2_O (Tables S1 and S2). The former showed a trigonal crystal system with a space group of R-3c while the latter showed a monoclinic crystal system and a space group of P2_1_/c. The crystal density of Al(NO_3_)_3_.9H_2_O is slightly higher than that of AlCl_3_.6H_2_O with values of 1.766 and 1.667 ρ_calc_ g/cm^3^, respectively. In term of similarities, both of the two single crystal structures consists of chains of Al(H_2_O)_6_^3+^ units, these units in AlCl_3_.6H_2_O single crystals are hydrogen bonded with chloride anions bridging between the chains. Whereas in Al(NO_3_)_3_.9H_2_O singles crystals these units are bonded with 3(NO_3_)^3−^ and 3 independent H_2_O molecules which are not coordinated to the Al^3+^ ions. The bond lengths of Al^3+^-O in AlCl_3_.6H_2_O and Al(NO_3_)_3_.9H_2_O singles crystals are quite similar with values of 1.881(2) and 1.868–1.896 Å, respectively.

The XRD patterns of as-prepared AlCl_3_.6H_2_O and Al(NO_3_)_3_.9H_2_O in diffractograms a and b in Figure S2, respectively. The XRD diffractogram of AlCl_3_.6H_2_O is in accordance with the standard PDF card of AlCl_3_.6H_2_O (44–1473), signifying that AlCl_3_.6H_2_O was successfully prepared in high purity^25^. The XRD diffractograms of Al(NO_3_)_3_.9H_2_O showed a successful transformation from the aluminum chloride phase (Figure S2(b)).

Figure S3 displays the XRD patterns of AC120, ACFL120, AC550 and ACFL550. Diffractograms AC120 and ACFL120 showed the diffraction lines that correspond to boehmite (γ-AlO(OH)) (JCDD 21-1307) at 2θ = 27, 34, 37, 44, 48, 64, and 72°, while the diffractograms of AC550 and ACFL550 showed the diffraction lines that correspond to γ-Al_2_O_3_ (JCDD 10-0425) at 2θ = 36, 45, 60, and 66°^19^. Therefore, the XRD results confirmed the formation of a boehmite phase from the aluminum chloride solution produced from the Al foil and consequently γ-Al_2_O_3_ phase after calcination at 550 °C.

Table 1 shows the surface areas and pore volume of ACFL120, AC120, ACFL550 and AC550. There is no significant difference in the surface area of ACFL120 and AC120, however, the pore volume in ACFL120 is approximately twice that of the AC120, 0.36 and 0.2 cm^3^ g^−1^, respectively. The crystallite size calculated by the Scherrer equation showed that ACFL550 and AC550 have relatively similar crystallite size (Table 1).

**Table 1:** Surface area (S_BET_ (m^2^ g^−1^)) and pore volume (cm^3^ g^−1^) for different acidic supports.

Support/Catalyst	S_BET_ (m^2^ g^−1^)	Crystallite size (nm)	Pore volume (cm^3^ g^−1^)	Total acidity^a^, A(sites.g^−1^)	Total acidity^b^, B(sites.m^−2^)	EDX results		
						Al	O	Cl
ACFL120	387	3.0	0.36	—	—	37	60.1	2.9
AC120	378	3.1	0.20	—	—	32.9	60.9	6.1
ACFL550	300	3.5	0.45	7.01	2.4	52.7	45.5	1.8
AC550	278	3.7	0.35	6.91	2.5	50.3	45.9	3.8

^a^Total acidity = A × 10^20^. ^b^Acid density = B × 10^18^.

**Figure 2:**
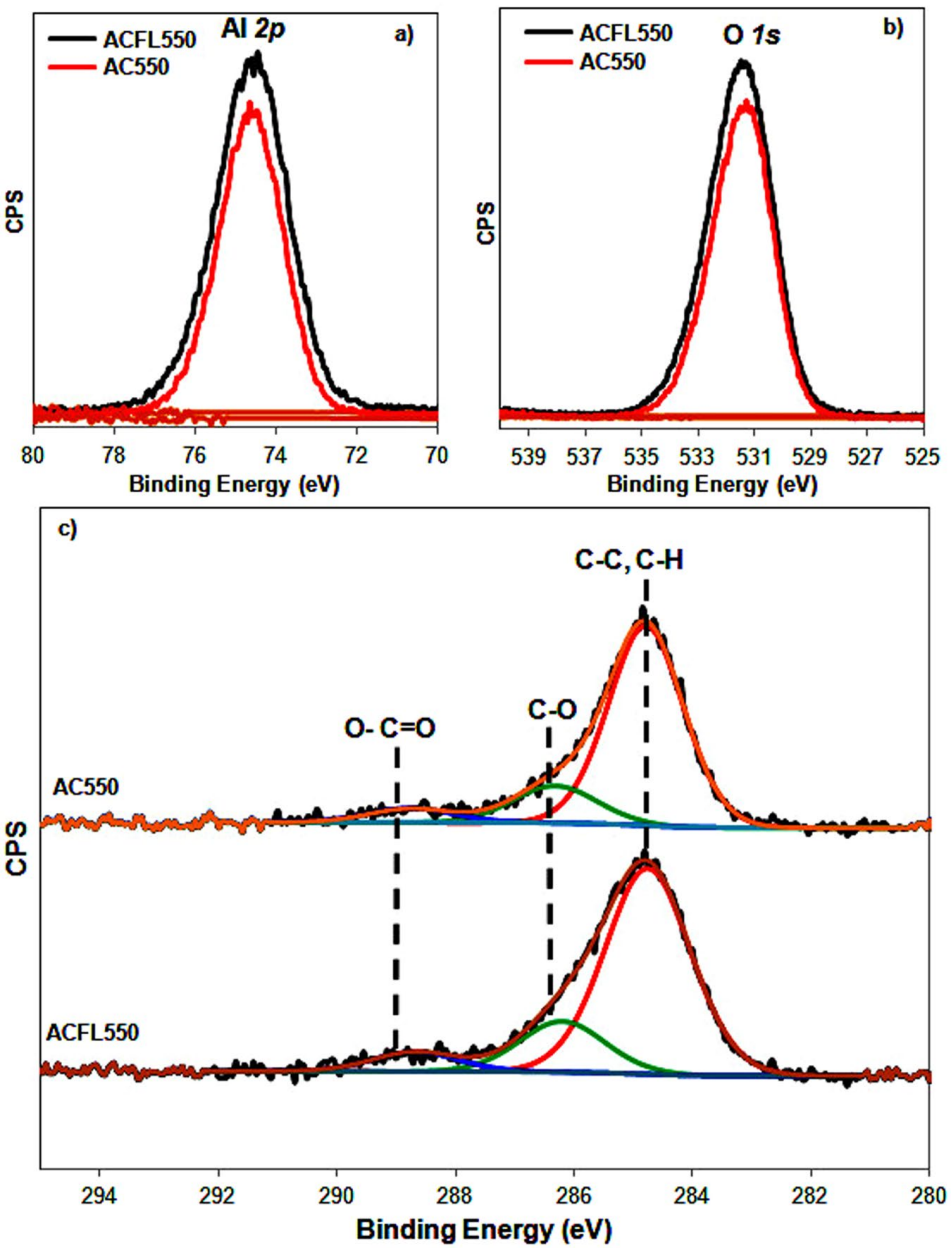
XPS of ACFL550 and AC550 catalysts (**a**) Al*2p*, (**b**) O*1s* and (**c**) adventitious carbon C*1s*.

XPS was performed to analyse the surface chemistry of ACFL550 and AC550 catalyst with core level binding energies were corrected using C *1s* peak at 284.8 eV as a charge reference. The full XPS spectra (survey) of ACFL550 matched that of AC550, confirming the successful preparation of γ-Al_2_O_3_ from Al foil (Figure S4)^26, 27^. Furthermore, the XPS spectra of Al *2p* (74.6 eV) and O *1s* (531.5 eV) along with the adventitious carbon are shown in Figure 2 ^28^. Again, the characteristic XPS peaks of γ-Al_2_O_3_ (Al *2p* and O*1s*) are similar in ACFL550 and AC550 catalysts (Figure 2(a,b))^27^. Since these two catalysts were left in the air, so the adventitious carbon is expected to present in the form of adsorbed carbonaceous species (CO, CO_2_ and CO_3_) and they showed similar trend of these species with adventitious carbon C-C at 284.8 eV, adsorbed CO/CO_2_ (C-O at 286.2 eV) and O-C=O at ~288.6 eV (Figure 2(c))^29^.

The successful formation of the single crystals (AlCl_3_.6H_2_O and Al(NO_3_)_3_.9H_2_O) was confirmed by the TGA analysis (Figure S5). The theoretical thermal decomposition of AlCl_3_.6H_2_O is 78.8% which can be calculated by Equation 1

1 $$2AlC{l}_{3}\cdot 6{H}_{2}O\to A{l}_{2}{O}_{3}+6HCl+9{H}_{2}O\,$$

The prepared AlCl_3_.6H_2_O showed a 78.8% weight loss which matched the theoretical calculation and Cui *et al*.^25^ work. Whereas Al(NO_3_)_3_.9H_2_O showed 85% weight loss which is comparable to the theoretical calculation by Equation 2 (85.9%).

2 $$2Al{(N{O}_{3})}_{3}\cdot 9{H}_{2}O\to A{l}_{2}{O}_{3}+6HN{O}_{3}+15{H}_{2}O\,$$

The suitable calcination temperature for obtaining γ-Al_2_O_3_ phase from boehmite was 550 °C^19^. Figure S6 shows the TGA curves of AC120, ACFL120, AC550 and ACFL550. It is apparent from the TGA curves of AC120 and ACFL120 that there are three weight loss steps corresponding to the phase transformations to γ-Al_2_O_3_ are observed. The first step started at 50 °C followed by two consecutive steps in the range 120–270 °C and 270–500 °C. After that, the rate of weight loss during heating up to 600 °C slowed with the formation of γ-Al_2_O_3_. The total % weight loss for AC120 and ACFL120 are 29.6 and 26.7%, respectively. As above, the XRD showed that boehmite was formed at 120 °C. Theoretically, the transformation of boehmite to γ-Al_2_O_3_ should be associated with 15% weight loss. The difference in weight loss of 14.6 and 11.7% can be attributed to the desorption of physisorbed water, the chlorine remaining in the bulk of the support as well as dehydroxylation occurring when boehmite transformed into γ-Al_2_O_3_^30^. The TGA curves of the AC550 and ACFL550 are apparently identical (Figure S6), which confirmed the formation of highly pure γ-Al_2_O_3_ support from the Al foil. The total % weight loss is 7.3% which can be attributed to desorption of physisorbed water, as there is no weight loss due to the phase change. These results are in agreement with our previous work and the XRD results^19^.

**Figure 3:**
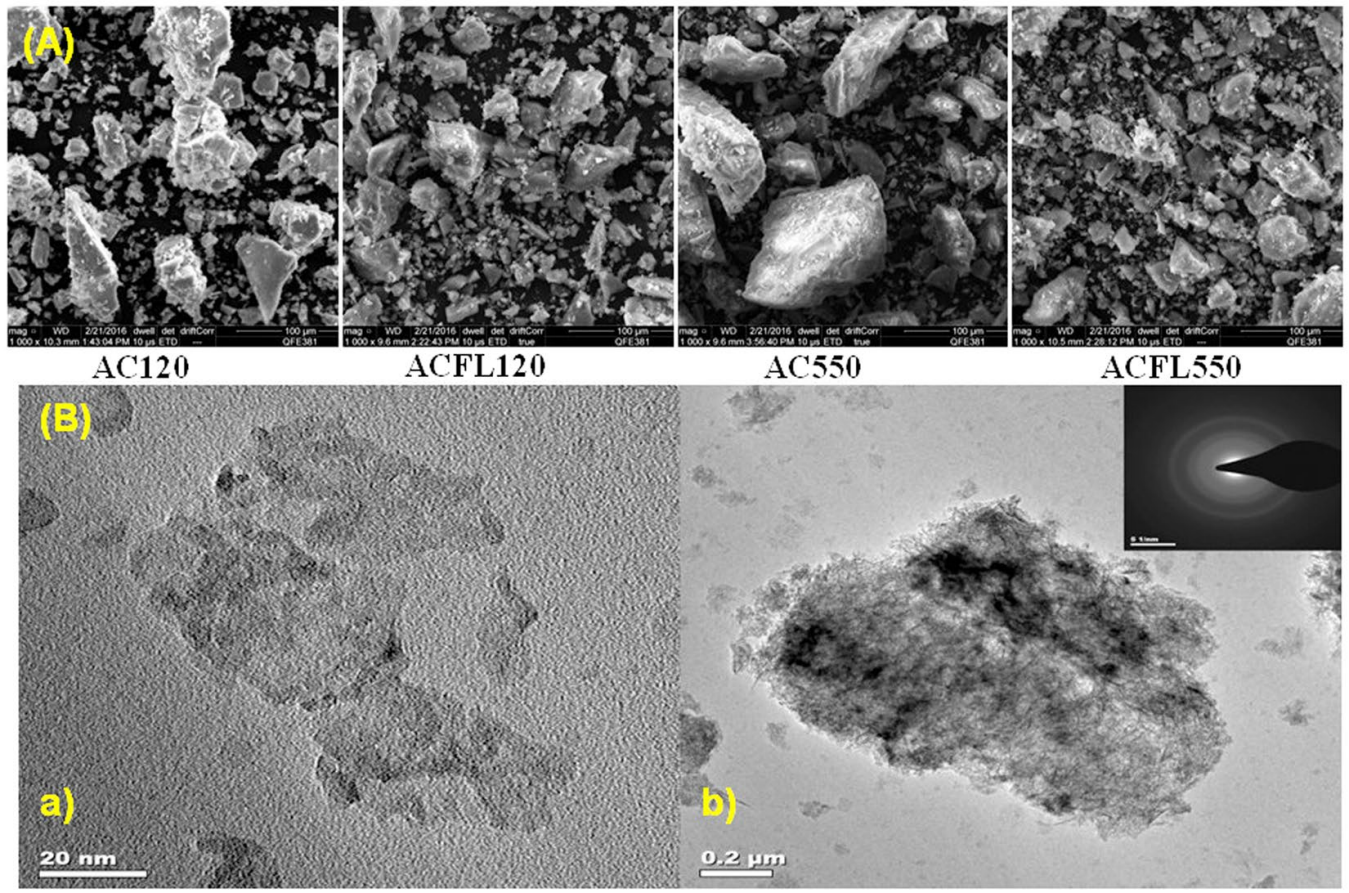
(**A**) SEM images of boehmite (γ-AlOOH) produced from commercial aluminium chloride (AC120) and aluminium foil waste (ACFL120) along with the γ-Al_2_O_3_ produced from commercial aluminium chloride (AC550) and aluminium foil waste (ACFL550). (**B**) (a) TEM overview image of the TEM-BF, (b) the acquired area of SAED pattern of the un-purified ACFL550 (γ-Al_2_O_3_ prepared from Al foil) with SAED pattern.

Figure 3A shows the SEM images of AC120, ACFL120, ACFL550 and AC550. There is no significant difference in the morphology of AC120 and ACFL120. The particles size in ACFL120 obviously smaller than that of AC120, especially at low magnification. Moreover, at high magnifications, AC120 tends to form big clusters while ACFL120 forms small tubes or fine particles (Figure S7). It can thus be concluded from the SEM that the morphology in the ACFL120 that prepared from Al foil is better than that of AC120. Apparently, there is a significant difference in the particles size in ACFL550 and AC550 (Figure 3A).

The EDX data of ACFL120 and AC120 are shown in Figure S8 and Table 1. It is worth noting that the % Cl content in ACFL120 is half than that of AC120 with 2.9 and 6.1 Cl%, respectively. It is well-known that the chlorine content can poison the catalyst and lead to severe catalytic deactivation^31^. Moreover, the % Al in ACFL120 is higher than that of AC120 with 37 and 32.9 Al%, respectively. Again, the chlorine content in AC550 is two times higher than ACFL550 with % Cl of 3.8 and 1.8%, respectively (Figure S8 and Table 1). These results can open doors for the support preparation with low chlorine content in the catalyst composition and consequently high catalytic performance, especially in acid-catalysed reactions.

TEM was used to investigate the primary particle size and morphology of the unpurified ACFL550, prepared by dissolving the Al foil in the acid followed by precipitation and calcination to form the γ-Al_2_O_3_. i.e. without any purification. Figure 3B(a) shows the bright field (BF) TEM. The High Angle Annular Dark Field Scanning Transmission Electron Microscope (HAADF-STEM) is shown in the supplementary (Figure S9). The ACFL550 particle sizes are obviously in the nano-scale of approximately 10 nm. Furthermore, the particles have a nano- rod shapes as seen in Figure 3B(b) and this is in line with the results of Mui *et.al*.^32^. The amorphous nature of ACFL550 nanoparticles was confirmed by the selected-area electron diffraction (SAED) as there were no diffraction spots observed in the SAED pattern, see a small window in Figure 3B(b) ^33^ and this is in agreement with the XRD results, Figure S3. The EDX spectrum in Figure (S9) showed that there are traces of Fe of 1.8% by weight, and 0.7% Fe by atomic percentage, this is may be due to the similar chemical properties of Al and Fe as they both precipitate in the hydroxide form using ammonia as precipitating agent. However, during the purification process, Fe was removed from the sample.

ICP-OES technique was used to detect the %Al in single crystals and γ-Al_2_O_3_ produced from Al foil and commercial precursors (ACFL550 and AC550). The theoretical %Al in AlCl_3_.6H_2_O and Al(NO_3_)_3_.9H_2_O single crystals are 11.18 and 7.19%, respectively. The detected %Al from commercial and Al foil were relatively the same (Table S3). Again the %Al in ACFL550 and AC550 were apparently similar with 45.79 and 45.21%, respectively.

**Figure 4:**
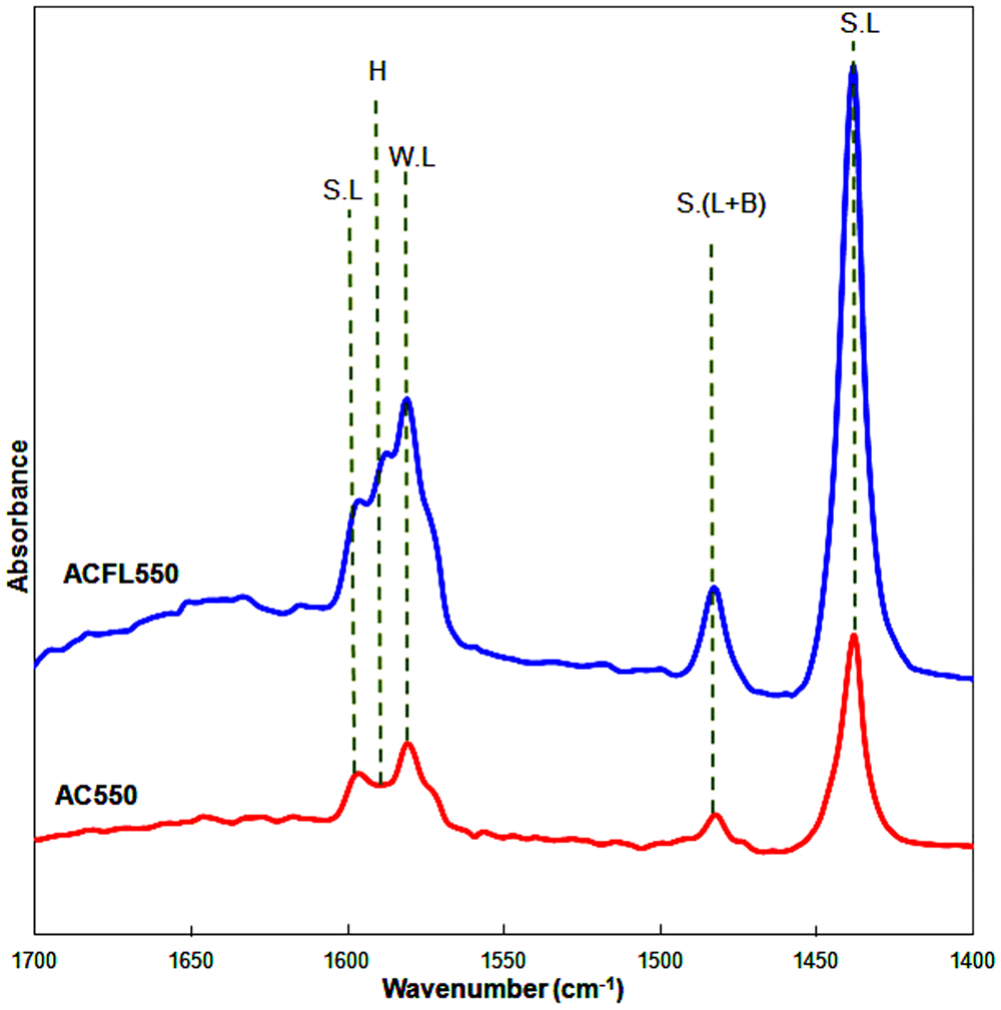
FTIR-Pyridine spectra in the regionof 1400–1700 cm^−1^ for ACFL550 and AC550 catalysts.

Figure 4 shows the FTIR-Pyridine of AC550 and ACFL550. Both catalysts showed mainly Lewis acidic sites in which there was no observation for the absorption band attributed to the Brönsted acidic site at 1550 cm^−1^ ^19^. The bands observed at 1440 and 1600 cm^-1^ are ascribed to the hydrogen bonded pyridine adsorbed on strong Lewis acid sites, while the band observed at 1582 is attributed to the presence of weak acidic sites. The absorption band at 1588 cm^−1^ is attributed to the hydrogen bound pyridine while the band at 1485 cm^−1^ is due to the pyridine adsorption on both Lewis and Brønsted acidic sites^19, 34^. It is obvious that Lewis acidic sites are responsible for the acidity for ACFL550 and AC550, while ACFL550 showed stronger acidity than that of AC550 (Figure 4). The TPD-Pyridine results showed that the total acidity of ACFL550 is higher than that of AC550 (Table 1), and this is in agreement with the FTIR-pyridine results.

### Catalyst applications in acid-catalysed reactions

**Figure 5:**
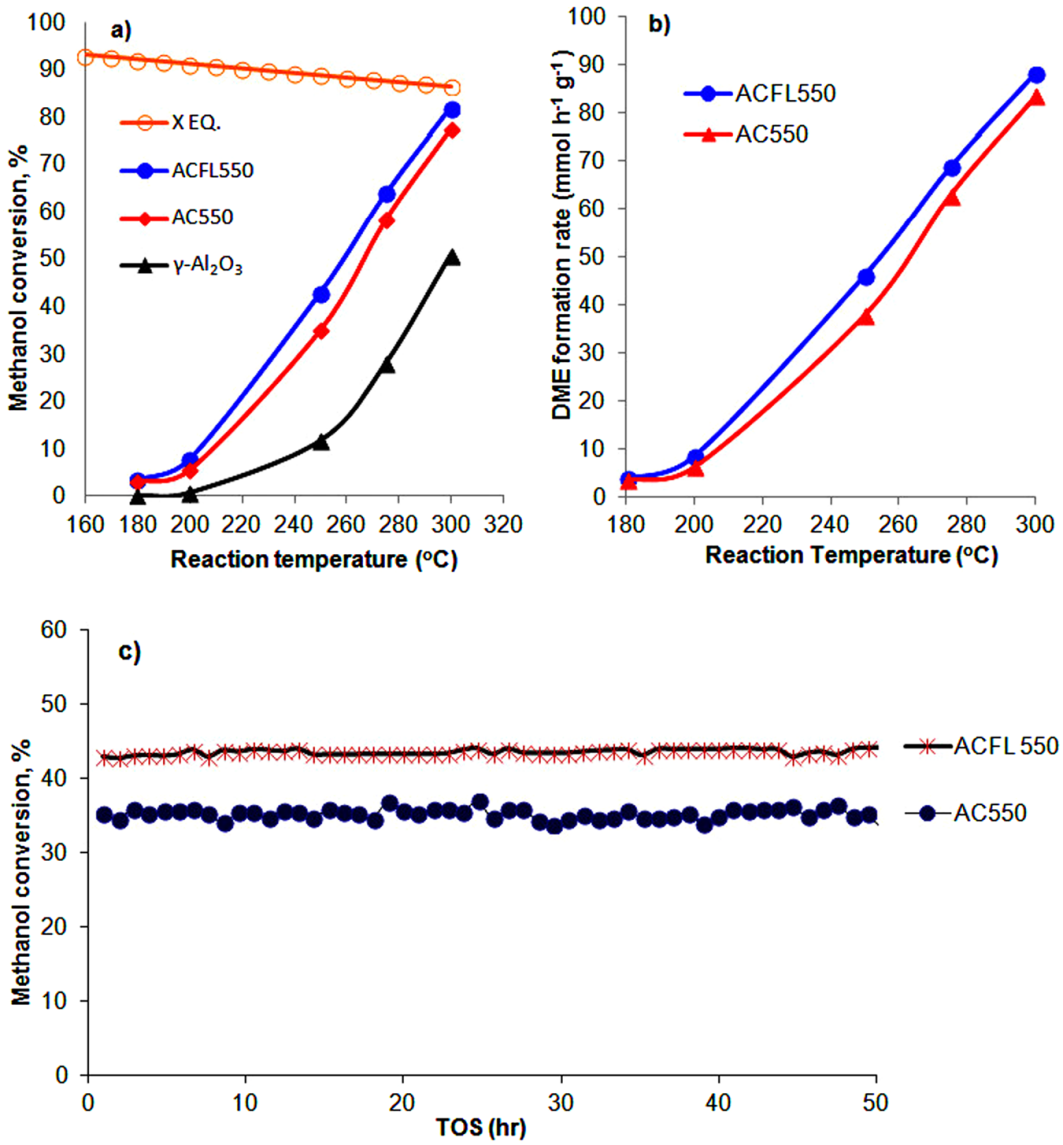
The catalytic conversion profiles of methanol dehydration of ACFL550, AC550 and the commercial γ-Al_2_O_3_ catalysts, (**a**) the % methanol conversion and (**b**) the DME formation rate (mmol h^−1^ g^−1^), under reaction conditions of 180–300 °C with a WHSV of 12.1 h^−1^ and (**c**) the time on stream test at 250 °C.

γ-Al_2_O_3_ is known as the commercial acid catalyst in the production of dimethyl ether (DME) from methanol dehydration. The dehydration occurs over a solid acid catalyst where its acidity plays a big role in product distribution. Over strong acidic sites, further dehydration will take place producing olefins. In order to suppress the dehydration to olefins reaction and increase the selectivity towards the DME, weak or moderate acidity catalysts are desirable. The most widely used catalysts are zeolites and γ-Al_2_O_3_^19^. Figure 5 shows the comparison of the catalytic activity of ACFL550 and AC550 which showed that ACFL550 is more active than that of AC550 with a close methanol conversion to the theoretical equilibrium (X.EQ) at a reaction temperature of 300 ºC with % conversion of 82% and 86.5%, respectively, followed by AC550 77.6% methanol conversion, (Figure 5(a)). This is in agreement with acidity results (TPD-pyridine and FTIR-Pyridine) which showed that ACFL550 has stronger Lewis acidic sites than that of AC550 catalyst (Figure 4 and Table 1). Moreover, the surface area and pore volume of ACFL550 are larger than that of AC550 as shown in Table 1. The comparison of the catalytic activity with the commercial γ-Al_2_O_3_ catalyst is shown in Figure 5(a). It is obvious that the commercial γ-Al_2_O_3_ was the least active catalyst compared with ACFL550 and AC550. At the reaction temperature of 275 °C, the catalytic activity of commercial γ-Al_2_O_3_ was less than half the activity of ACFL550 and AC550 catalysts. This is due to its lowest surface area (117 m^2^ g^−1^) and surface total acidity (4.11 × 10^20^ sites. g^−1^) compared with the highest surface area for ACFL550 (300 m^2^ g^−1^) and surface acidity of 7.01 × 10^20^ sites. g^−1^ as seen in Table 1.

Figure 5(b) shows that the DME formation rate (mmol h^−1^ g^−1^) of ACFL550 is apparently higher than that of AC550 catalyst which is in agreement with the catalytic conversion in Figure 5(a). This concludes that ACFL550 has a superior surface structure and acidity and lower chlorine content, which makes it a promising acidic catalyst in the methanol dehydration reaction with high DME formation rate.

The stability test of ACFL550 and AC550 catalysts was studied for 50 hours at 250 °C. Generally, γ-Al_2_O_3_ is characterised with weak and medium acidic sites and accordingly is preferable in methanol dehydration reaction due to it providing good stability with low carbon and by- product formation^35, 36^. Figure 5(c) shows the methanol conversion as a function of time on stream; apparently, both the two catalysts (ACFL550 and AC550) are stable during the methanol dehydration reaction.

One of the applications of the modified γ-Al_2_O_3_ is in the total methane oxidation (TMO) or partial methane oxidation (PMO) as an active support^31, 37^. Recently, the bimetallic PdPt loaded on a dual component support showed superior activity in the TMO reaction of palladium being the most active component in the catalyst composition. It is well known that the acidic support is crucial for the TMO reaction as it makes the Pd metal more electron deficient and enhances the re-oxidation of Pd(0) during Pd(0)/PdO redox cycle and consequently improve the catalytic activity^31^. It is obvious that the catalysts that have acidic support component (5Pd-ACFL and 5Pd-AC) showed higher catalytic activity than that of the mono-component support (93% TiO_2_) without acidic support (Figure S10). The lower catalytic activity in the 93% TiO_2_ catalyst is due to the absence of the acidic support in the catalyst composition. The catalytic activity of 5Pd-ACFL (derived from Al foil) catalyst was apparently higher than that of 5Pd-AC (derived from the Al chloride precursor) and 93% TiO_2_ catalysts with T_50%_ at 303, 321 and 340 ºC, respectively. The superior catalytic activity of 5Pd-ACFL than that of 5Pd-AC may be due to the stronger Lewis acidic sites in the ACFL550 than that of AC550 supports as discussed earlier. Furthermore, the chlorine contents are known to block the active sites on the catalyst surface and consequently lead to catalytic deactivation^38–40^. EDX analysis in Table 1 showed that the % Cl in ACFL550 is half that of AC550 with 1.8 and 3.8, respectively. Thus it is not surprising that 5Pd-ACFL showed a higher catalytic activity than that of 5Pd-AC in the TMO reaction.

## Conclusion

Herein, we propose a novel eco-friendly preparation route firstly to obtain two crystalline aluminum single crystals from aluminum foil waste using a simple preparation method. The produced AlCl_3_.6H_2_O and Al(NO_3_)_3_.9H_2_O were characterized by single crystal XRD and showed two different crystal structure, a trigonal (R-3c) and a monoclinic (P2_1_/c), respectively. A green synthetic route was followed to prepare nano-mesoporous γ-Al_2_O_3_ from AlCl_3_.6H_2_O designated as ACFL550, which showed higher surface area, larger pore volume, stronger acidity and surface structure compared with the γ-Al_2_O_3_ that produced from the commercial AlCl_3_ precursor, AC550. EDX analysis showed that ACFL550 had half the chlorine content (Cl %) relative to AC550, which makes ACFL550 a promising catalyst in acid catalysed reactions. Pure and modified ACFL550 and AC550 were applied in acid catalysed reactions, the dehydration of methanol to dimethyl ether and the total methane oxidation reactions, respectively. It was found that ACFL550 showed higher catalytic activity than AC550 due to its superior surface structure and acidity. The modified ACFL550 showed higher activity in the TMO reaction with T_50%_ at 303 ºC. These results open doors for the preparation of highly active and well-structured nano-mesoporous alumina catalyst/support from aluminum foil waste and its application in acid-catalysed reactions.

## Methods

### Materials and methods

The Al foil waste was collected from the laboratories in David Kier building at Queen’s University Belfast. The chemicals used in the present study were all of analytical grade and supplied by Aldrich, UK. These included aluminum nitrate nonahydrate Al(NO_3_)_3_·9H_2_O, anhydrous aluminum chloride (AlCl_3_) and ammonia solution (35%). The commercial γ-Al_2_O_3_ (BET = 117 m^2^g^−1^) was supplied from Alfa Aesar, used after crushing the pellets in particle size of 250–425 µm, designated as γ-Al_2_O_3_ and was used without any further purification. All gases, He, CH_4_, 20% O_2_/Ar and Ne were obtained at 100% purity from BOC gases, UK.

### Catalyst preparation

#### Preparation of AlCl_3_.6H_2_O and Al(NO_3_)_3_.9H_2_O single crystals

The required amount of the foil waste was dissolved in a 6 M HCl solution to form the aluminum chloride solution. Then the solution was kept under a desiccator until crystalline AlCl_3_.6H_2_O formed. Then these crystals were dissolved in deionized water and filtered to remove any impurities. After that, the single crystals were purified by recrystallization using 20 wt% deionized water and heated the mixture up till all these crystal dissolved and stirred for 30 min then kept under desiccator to re-crystallize, and the remaining water was removed. This step was repeated three times to obtain high purity AlCl_3_.6H_2_O.

The crystalline Al(NO_3_)_3_.9H_2_O was prepared from the AlCl_3_ solution prepared by dissolving the foil in the 6 M HCl solution, then a required amount of 15.6 M HNO_3_ was added then followed by heating to form the Al(NO_3_)_3_.9H_2_O solution and finally was kept under desiccator to form a pure crystals (Figure S1(a)). The purification procedure was done as described earlier. As the preparation of Al(NO_3_)_3_.9H_2_O lead to emissions of NOx and chlorine gases (Equation 3), so we decided to prepare the γ-Al_2_O_3_ from AlCl_3_ as this preparation route is a “greener” route without any emissions or waste during the preparation procedures.

3 $$3C{l}^{-1}+HN{O}_{3}\to NOCl+C{l}_{2}+2{H}_{2}O\,$$

#### Preparation *of* γ-Al_2_O_3_ from AlCl_3_.6H_2_O

The preparation of the γ-Al_2_O_3_ support from AlCl_3_.6H_2_O has been described in previous work^19^. AlCl_3_ (the commercial one or that prepared from the aluminum foil) was firstly dissolved in deionised water with continuous stirring at 60 °C. When the solution was completely mixed and heated up to 100 °C, an ammonia solution (35%) was added dropwise resulting in precipitation. After precipitation completed, stirring continued for a further 12 hrs at room temperature (approximately at 18 °C). The pale off-white precipitate was filtered, washed, and dried at 120 °C overnight. The dried precipitates designated as ACFL120 and AC120 for those prepared from the aluminum foil and the commercial AlCl_3_, respectively. The precipitate was then calcined at 550 °C for 4 hrs in a muffle furnace. The alumina supports designated as ACFL550 and AC550 for those prepared from the aluminum foil and the commercial AlCl_3_, respectively.

#### Preparation of modified ACFL550 and AC550 for the TMO reaction

The preparation of a bimetallic PdPt loaded on a dual component support (TiO_2_/ZSM-5(80)) has been described in previous work^31^. The catalysts were prepared using a wet impregnation method with the aid of sonication with a catalyst composition of 5% Pd+ 2% Pt + 17.5%TiO_2_ + 75.5% Al_2_O_3_ (ACFL550 or AC550). Pure supports ACFL550 or AC550 were placed in a vial and the mass of metal precursor solution or slurry, required to give 5 wt% palladium and 2 wt% platinum loading was added followed by TiO_2_ to give 17.5 wt% in deionized water (5 mL). The mixture was sonicated at 80 °C (Crest ultrasonic bath model 200 HT), under a 45 kHz frequency for 3 h. All mixtures were dried at 120 °C overnight in an oven before being calcined in air at 500 °C for 4 h with a heating ramp of 2 °C min^−1^. Finally, the produced catalysts were further washed with hot deionised water until no chlorine was detected using AgNO_3_. The washed catalysts were calcined in air at 250 °C for 3 h with a heating rate of 2 °C min^−1^ and designated as 5Pd-ACFL and 5Pd-AC for the modified catalysts derived from Al foil and Al chloride precursor, respectively. For a comparison, the catalyst without acidic support i.e. 5%Pd+ 2%Pt+93%TiO_2_ was prepared as discussed earlier and designated as 93% TiO_2_.

### Catalyst Characterization

X-ray Crystallography was performed for the structure of the AlCl_3_.6H_2_O and Al(NO_3_)_3_.9H_2_O. Suitable single crystals were cut from larger ones and mounted on a SuperNova, dual source (Cu K_α_ X-ray), EosS2 diffractometer. The crystal was kept at 100(10) K during data collection. Using Olex2^41^, the structure was solved with the ShelXT^42^ structure solution program using Direct Methods and refined with the ShelXL^42^ refinement package using Least Squares minimization.

Powder X-ray diffraction (XRD) was carried out using a PANalytical X’Pert Pro X-ray diffractometer. This diffractometer was equipped with a CuK_α_ X-ray source with a wavelength of 1.5405 Ǻ. The diffractograms were collected up to 2θ = 80°. The X-ray tube was set at 40 kV and 40 mA. Peaks were selected and compared to diffraction patterns in the software library. The crystallite size was calculated according to Scherrer equation.

4 $${\rm{Crystallite}}\,{\rm{size}}=\frac{0.9\times {\rm{\lambda }}}{{\rm{d}}\,\cos \,{\rm{\theta }}}\,$$

λ = 1.54060 Å (in the case of CuKa1) so, 0.9 × λ = 1.38654, d = the full width at half maximum intensity of the peak (in Rad).

Brunauer-Emmett-Teller (BET) analysis was performed using a Micromeritics ASAP 2020 system. BET surface area and pore volume were measured by N_2_ adsorption and desorption isotherms at liquid nitrogen temperature (−196 °C).

XPS was performed in a Kratos AXIS ULTRA spectrometer using monochromatic Al Kα radiation of energy 1486.6 eV. High-resolution spectra were taken at fixed pass energy of 20 eV, 0.05 eV step size and 100 ms dwell time per step. Surface charge was efficiently neutralized by flooding the sample surface with low energy electrons. Core level binding energies were corrected using C *1s* peak at 284.8 eV as charge reference. For construction and fitting of synthetic peaks of high-resolution spectra, a mixed Gaussian-Lorenzian function with a Shirley type background subtraction were used.

Scanning Electron Microscopy (SEM) was carried out on a FEI Quanta 250 FEG MKII with a high-resolution environmental microscope (ESEM) using XT Microscope Control software and linked to an EDX detector. The EDX used was a 10 mm^2^ SDD Detector-x-act from Oxford Instruments which utilizes Aztec® EDS analysis software. Both systems used the same chamber.

The chlorine content was measured using Oxygen Flask analysis by subjecting the sample to combustion in an oxygenated flask containing water, followed by shaking; in which chlorine dissolved in water forming HCl solution, the latter solution was titrated with 0.02 M Hg_2_NO_3_ to obtain the percentage of chlorine.

The acidity populations over the surface of catalysts, under investigation, were measured thermogravimetrically using the adsorption of pyridine as a probe molecule. Small portions (50 mg) of each sample were pre-heated at 250 °C for 2 h in the air before the exposure to the probe molecule. 15–20 mg of pyridine–covered samples were subjected to TG analysis on heating up to 600 °C (at 20 °C/min heating rate) in dry N_2_ (flow rate = 40 ml/min). The mass loss due to desorption of pyridine from the acidic sites was determined as a function of total surface acidity as sites.g^−1^_cat_. Determination of the concentration of Lewis and Brönsted acid sites on the pyridine-covered ACFL550 and AC550 samples was performed by Perkin Elmer-Spectrum 100, FT-IR spectrometer.

Inductively coupled plasma optical emission spectrometry (ICP-OES) was used to determine the elemental analysis of the aluminum single crystals and γ-Al_2_O_3_ samples derived from Al foil and commercial precursor. The Al single crystals were dissolved in deionized water, while the γ-Al_2_O_3_ samples were soluble in aqua regia. The solution of each sample was analyzed with an ICP Optical Emission Spectrometer (Optima 4300 DV, Perkin-Elmer).

### Catalyst application in acid catalysed reactions

#### Methanol dehydration to dimethyl ether

The activity testing for methanol dehydration was carried out in an isothermal fixed-bed reactor made of stainless steel (6 mm OD). The catalyst bed consisted of 100 or 200 mg (250–425 µm) of a catalyst placed in between two plugs of quartz wool. Aera mass flow controllers were used to control the flow of He to the reactor. The liquid methanol was injected by patented Cheminert® M Series liquid handling pump. A stable flow of methanol vapour to the reactor was established by passing the combined flow He and methanol through a saturator system. The evaporation chamber was kept at 150 °C. In order to prevent any condensation, all of the lines were heated to 150 °C. This mixture was then fed to the fixed bed reactor. The reaction conditions used 20% methanol under atmospheric pressure and over a temperature range from 180–300 °C. The total flow rate was 100 cm^3^ min^−1^. Before the reaction, the catalyst was activated in a stream of pure He at 325 °C for 0.5 h under atmospheric pressure. Then, the methanol and He mixture were fed to the reactor and samples analyzed by gas chromatography (Perkin-Elmer 500) equipped with thermal conductivity and flame ionization detectors.

#### The total methane oxidation (TMO) reaction

The activity testing for the TMO was carried out in an isothermal fixed-bed reactor made of stainless steel (6 mm OD) at atmospheric pressure. The catalyst bed consisted of 50 mg of catalyst, pelletized and sieved to between 250–425 µm, placed between two plugs of quartz wool. Aera mass flow controllers were used to control the flow of Ar, CH_4_, O_2_/Ar and Ne. Prior to the catalytic tests, each catalyst was oxidized under a flow of 5% O_2_/Ar at a flow rate of 40 mL min^−1^ at 500 °C for 2 h with a heating rate 5 °C min^−1^. The reaction gas mixture, which consisted of 0.5% CH_4_, 15% O_2_, 5% Ne and 79.5% Ar, was then fed to the fixed bed reactor with the total flow maintained at 83.3 mL min^−1^, giving a GHSV of 100,000 mL g^−1^ h^−1^. The products were analyzed by on-line gas chromatography using a Perkin Elmer 500 GC equipped with a Haysep column and TCD and FID detectors. The internal standard was 5% Ne in the feed. The reaction temperature was monitored throughout by a thermocouple placed in the centre of the catalyst bed. Under the conditions used no exotherm was observed.

## Electronic supplementary material

Supplementary

the supplementary video 1

the supplementary video 2
