# Supplementary material for: A Facile Green Synthetic Route for the Preparation of Highly Active γ-Al2O3 from Aluminum Foil Waste
Source: Sci Rep. 2017 Jun 15;7:3593. doi: 10.1038/s41598-017-03839-x (PMC5472618; doi:10.1038/s41598-017-03839-x)
Supplement: Supplementary file 1 — Supplementary [file 41598_2017_3839_MOESM1_ESM.doc]

Electronic Supplementary Information

For

**A Facile Green Synthetic Route for the Preparation of Highly Active γ-Al_2_O_3_ from Aluminum Foil Waste**

*Ahmed I. Osman^a,b^*, Jehad K. Abu-Dahrieh^a^*,* *Mathew McLaren^c^, Fathima Laffir^d^, Peter Nockemann^a^, David Rooney^a^*

^a^ School of Chemistry and Chemical Engineering, Queen's University, David Keir Building, Stranmillis Road, Belfast BT9 5AG, Northern Ireland, United Kingdom
^b^ Chemistry Department, Faculty of Science - Qena, South Valley University, Qena 83523 – Egypt.

^c^Centre for Nanostructured Media, School of Mathematics and Physics, Queen's University Belfast, BT7 1NN, UK.

^d^ Department of Chemical and Environmental Sciences, Materials and Surface Science Institute, University of Limerick, Limerick, Ireland

* Corresponding author

*Jehad K. Abu-Dahrieh, Ahmed I. Osman*

E-mail: [j.abudahrieh@qub.ac.uk](mailto:j.abudahrieh@qub.ac.uk), aosmanahmed01@qub.ac.uk

Address: School of Chemistry and Chemical Engineering, Queen's University Belfast, David Keir Building, Stranmillis Road, Belfast BT9 5AG, Northern Ireland, United Kingdom

Fax: +44 2890 97 4687

Tel.: +44 2890 97 4412

**Supplementary video 1:** Al chloride single crystal

**Supplementary video 2:** Al nitrate single crystal

**X-ray single crystal supplementary information**

**Crystal Data** for HOAlCl (*M*=79.44 g/mol): trigonal, space group R-3c (no. 167), *a* = 11.8260(2) Å, *c* = 11.9064(3) Å, *V*= 1442.07(5) Å^3^, *Z* = 27, *T* = 291.58(10) K, μ(Cu Kα) = 9.475 mm^-1^, *Dcalc* = 1.6679 g/cm^3^, 2741 reflections measured (14.98° ≤ 2θ ≤ 144.7°), 321 unique (*R*_int_ = 0.0503, R_sigma_ = 0.0193) which were used in all calculations. The final *R*_1_ was 0.0960 (I>=2u(I)) and *wR*_2_ was 0.2350 (all data).

**Crystal Data** for Al(NO_3_)_3_.9H_2_O (*M*=58.00 g/mol): monoclinic, space group P2_1_/c (no. 14), *a* = 13.85684(14) Å, *b* = 9.45095(10) Å, *c* = 10.87930(13) Å, *β* = 95.4618(9)°, *V*= 1418.29(3) Å^3^, *Z* = 26, *T* = 123.90(10) K, μ(CuKα) = 4.945 mm^-1^, *Dcalc* = 1.766 g/cm^3^, 10835 reflections measured (11.35° ≤ 2θ ≤ 145.454°), 2778 unique (*R*_int_ = 0.0179, R_sigma_ = 0.0126) which were used in all calculations. The final *R*_1_ was 0.0558 (I > 2σ(I)) and *wR*_2_ was 0.2261 (all data).


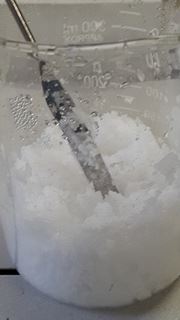


**a)**

**
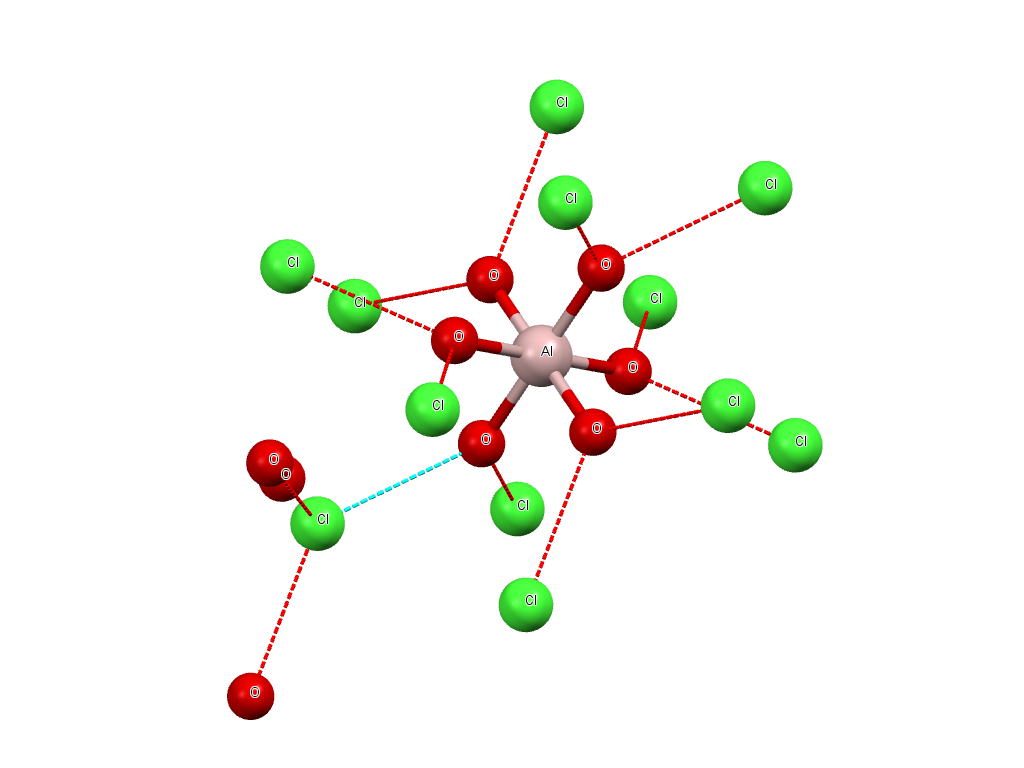
**

b)

**
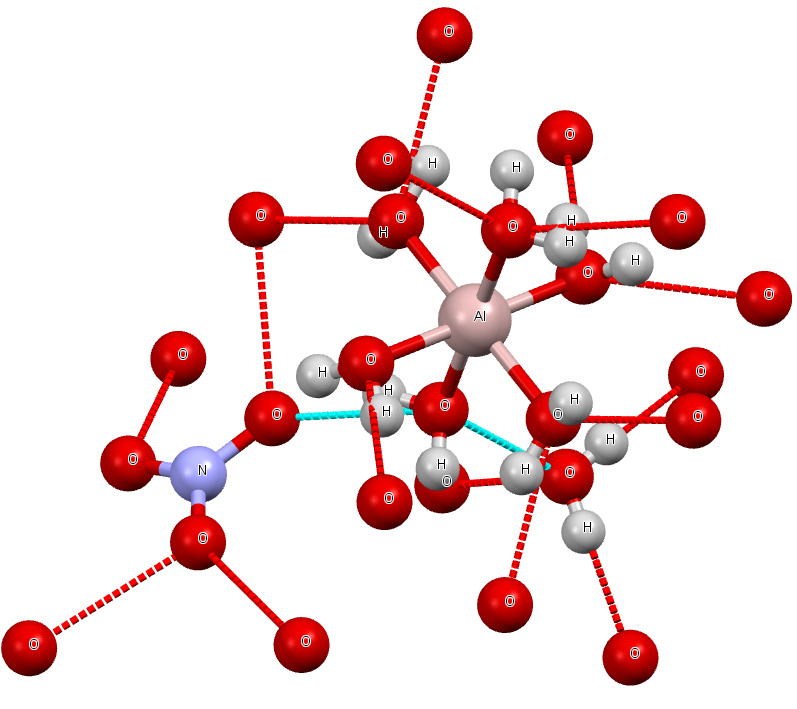
**

c)

**Figure S1:** a) Aluminium nitrate single crystal derived from the Al foil, b) and c) images show the hydrogen bond formation in the x-ray single crystal of homemade aluminium chloride hexahydrate and aluminium nitrate nonahydrate, respectively.

**
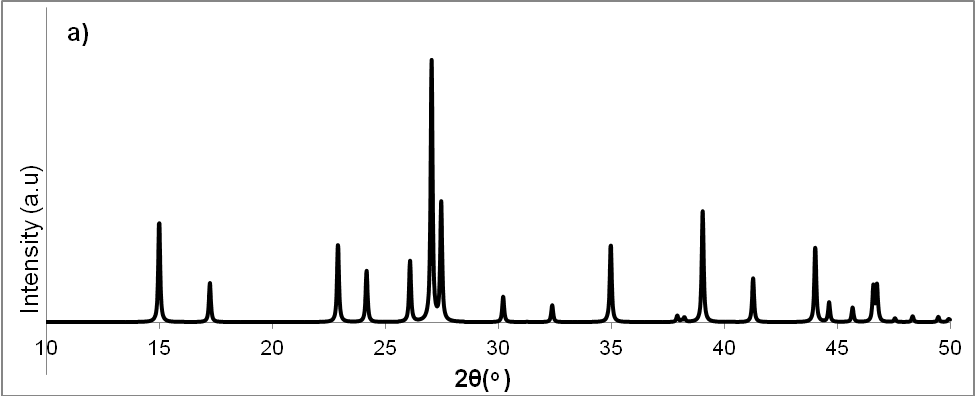
**
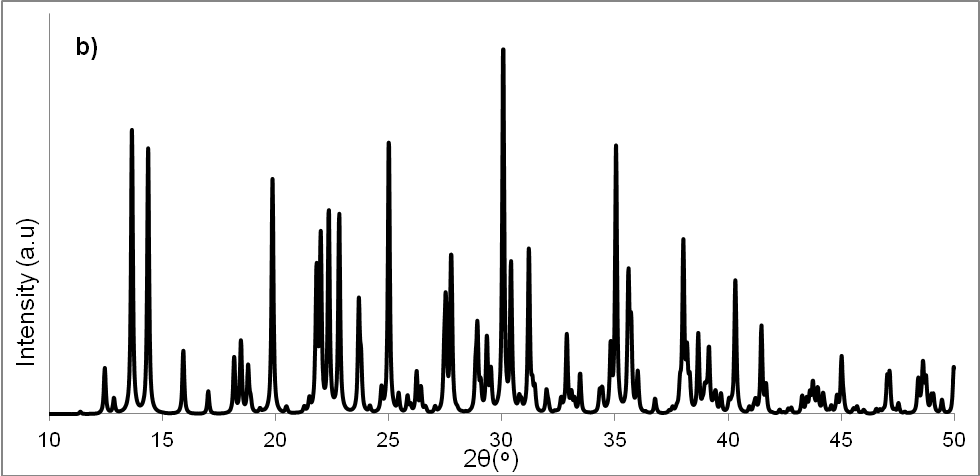


**Figure S2:** X-ray powdered of a) aluminium chloride hexahydrate and b) aluminium nitrate nonahydrate.


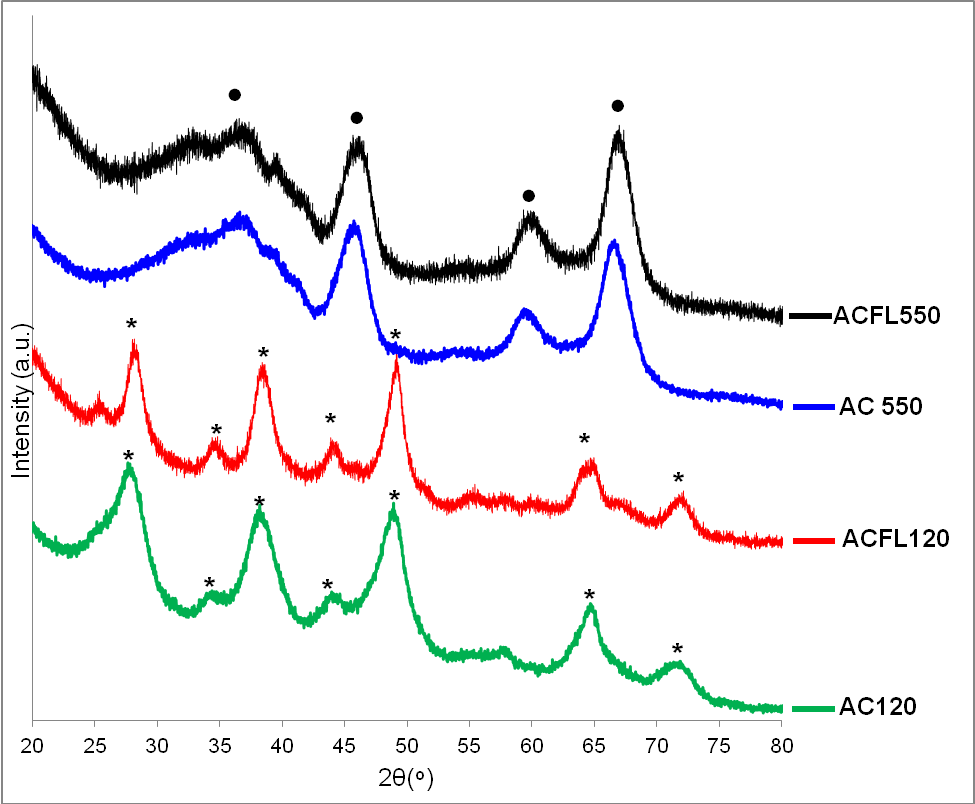


* boehmite

● γ-Al_2_O_3_

**Figure S3**: XRD patterns of Boehmite (γ-AlOOH) and γ-Al_2_O_3_ produced from commercial aluminium chloride (AC120 and AC550) and aluminium foil waste (ACFL120 and ACFL550).


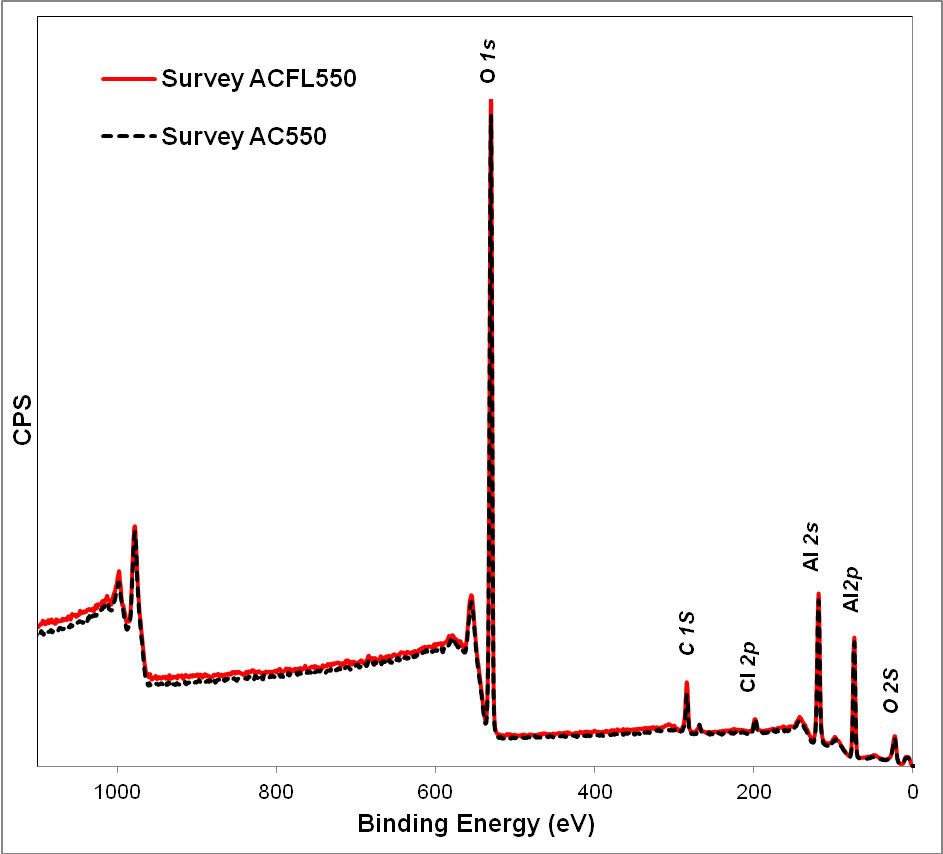


**Figure S4:** XPS survey of ACFL550 and AC550 catalysts at binding energy range of 0-1000 Ev.


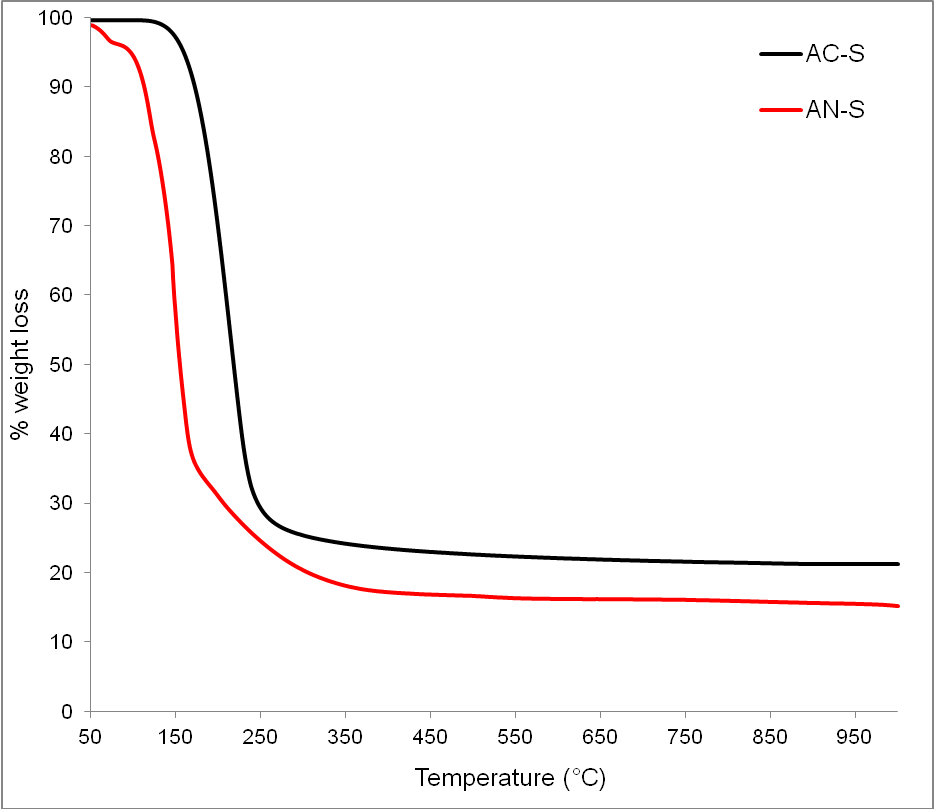


**Figure S5:** TGA curves of aluminium chloride and nitrate single crystals derived from the Al foil.


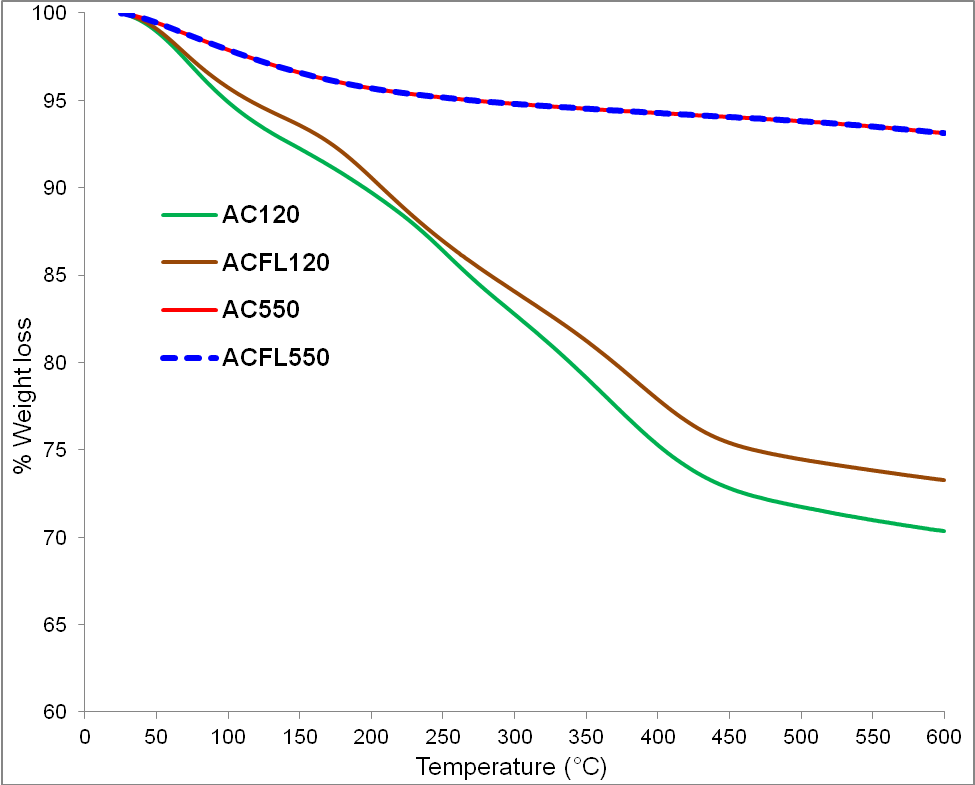


**Figure S6**: TGA curves of Boehmite (γ-AlOOH) and γ-Al_2_O_3_ produced from commercial aluminium chloride (AC120 and AC550) and aluminium foil waste (ACFL120 and ACFL550).

| 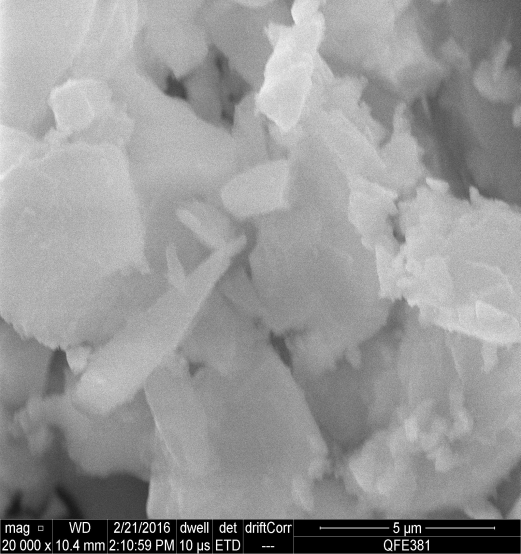  **AC120** | 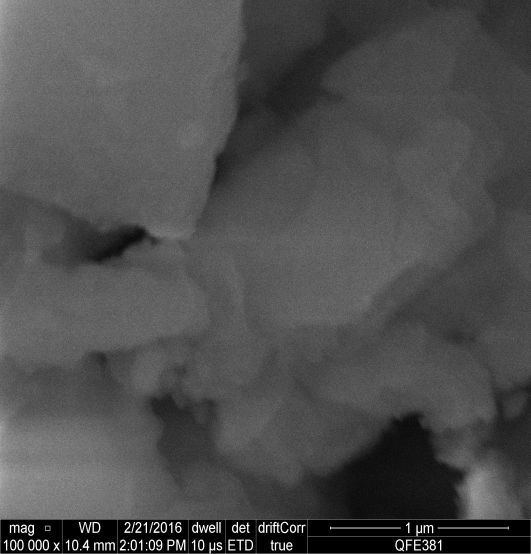 | |
| --- | --- | --- |
| 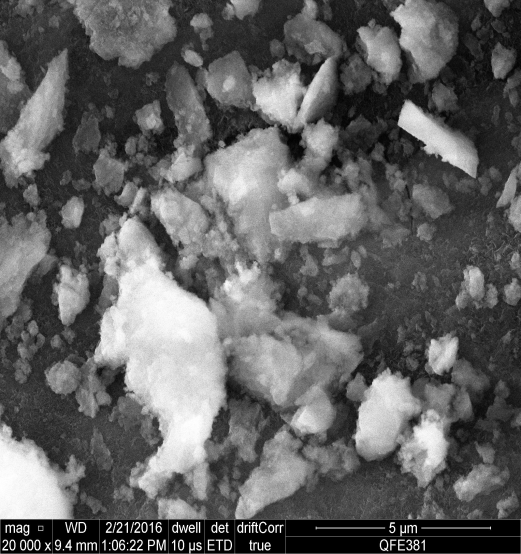  **ACFL120** | 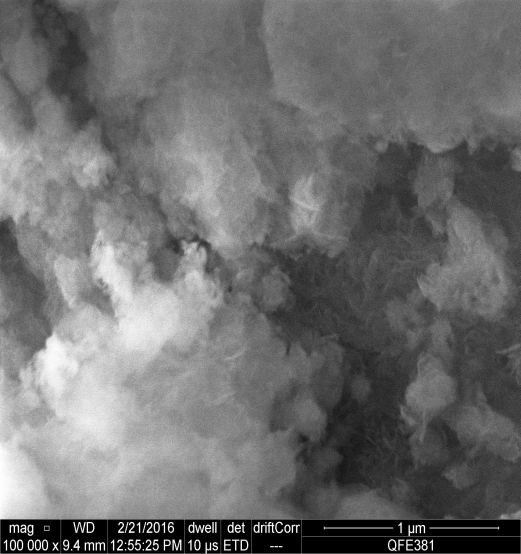 | |
| 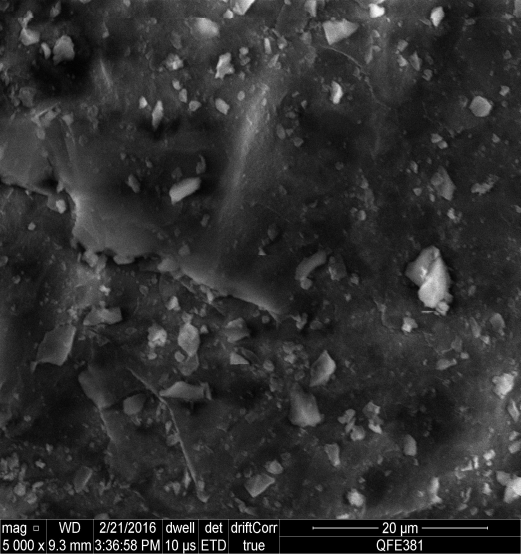  **AC550** | 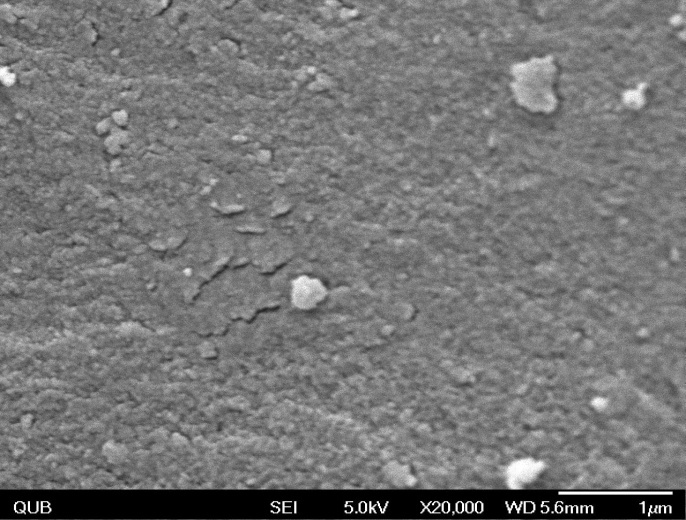 |  |
| 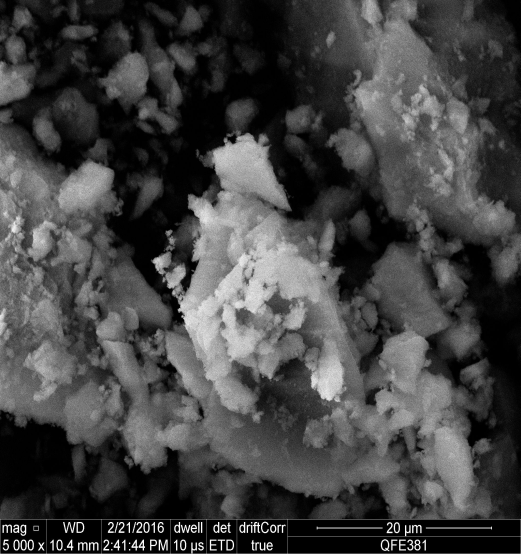  **ACFL550** | 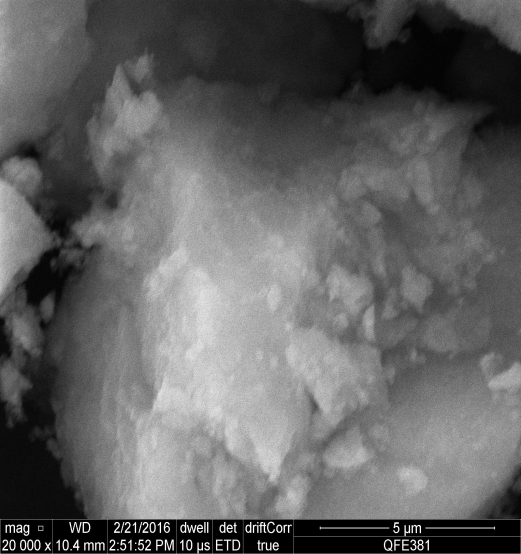 |  |

**Figure S7**: SEM images of Boehmite (γ-AlOOH) produced from commercial aluminium chloride (AC120) and aluminium foil waste (ACFL120) along with the γ-Al_2_O_3_ produced from commercial aluminium chloride (AC550) and aluminium foil waste (ACFL550).

| 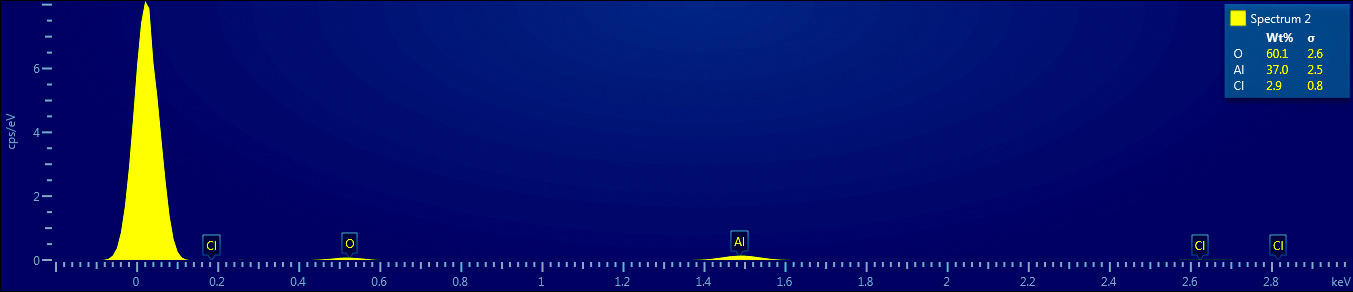  **a)** |
| --- |
| 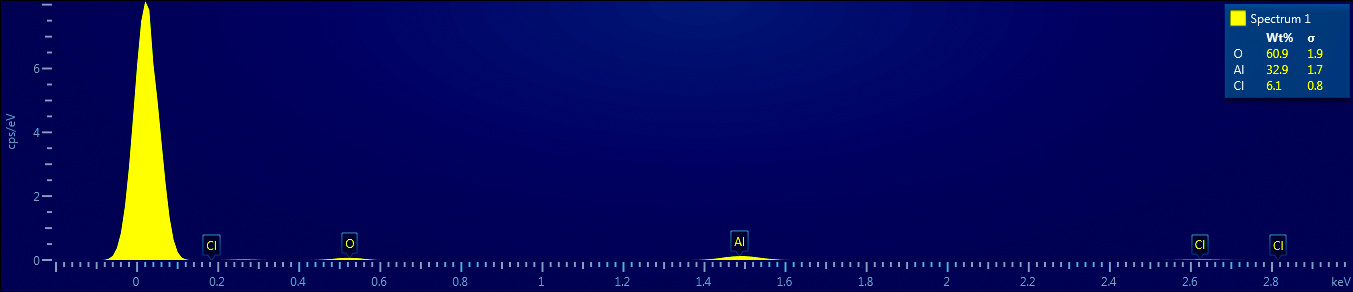  **b)** |
| 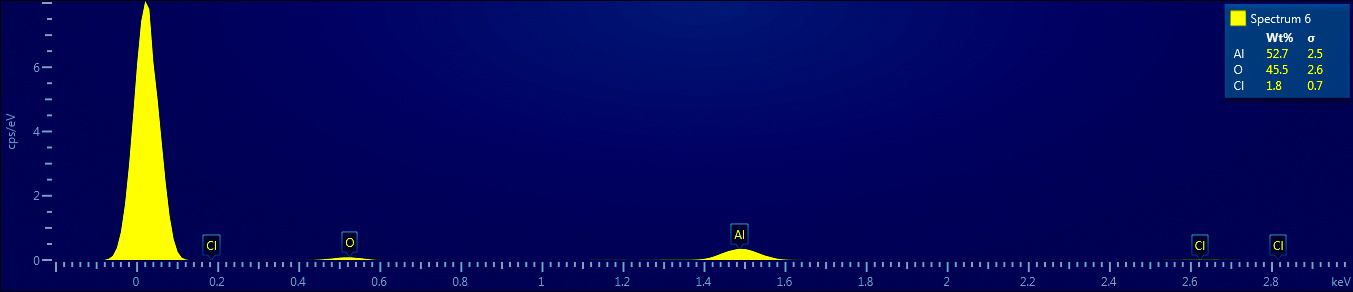  **c)** |
| 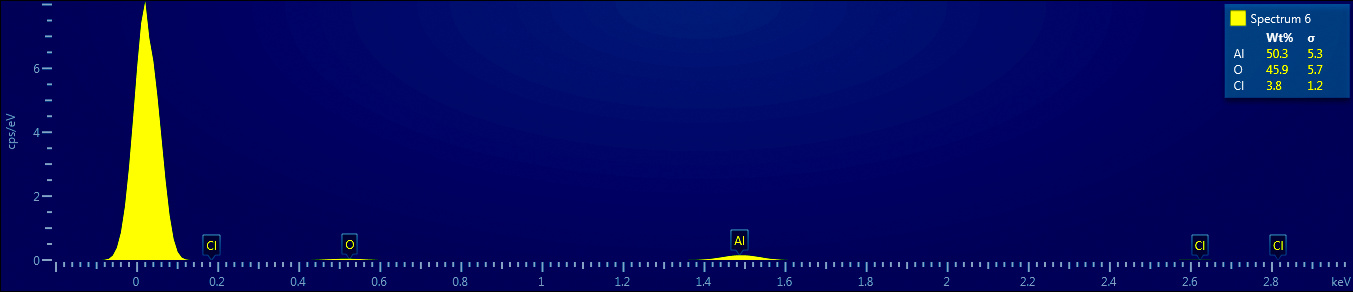  **d)** |

**Figure S8**: EDX data of a) ACFL120, b) AC120, c) ACFL550 and d) AC550 catalysts.

**a)**

**b)**


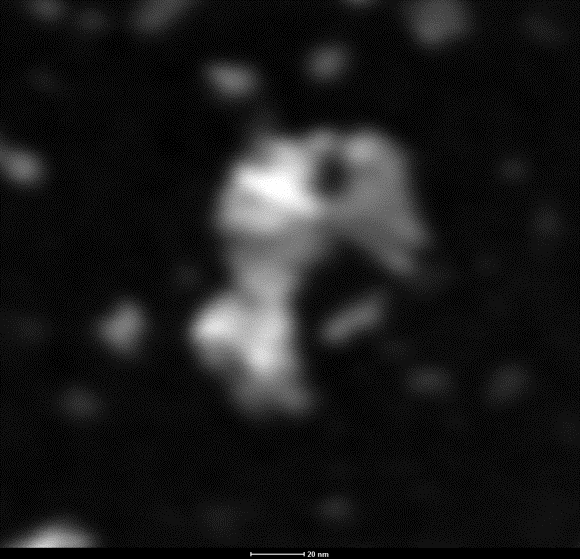


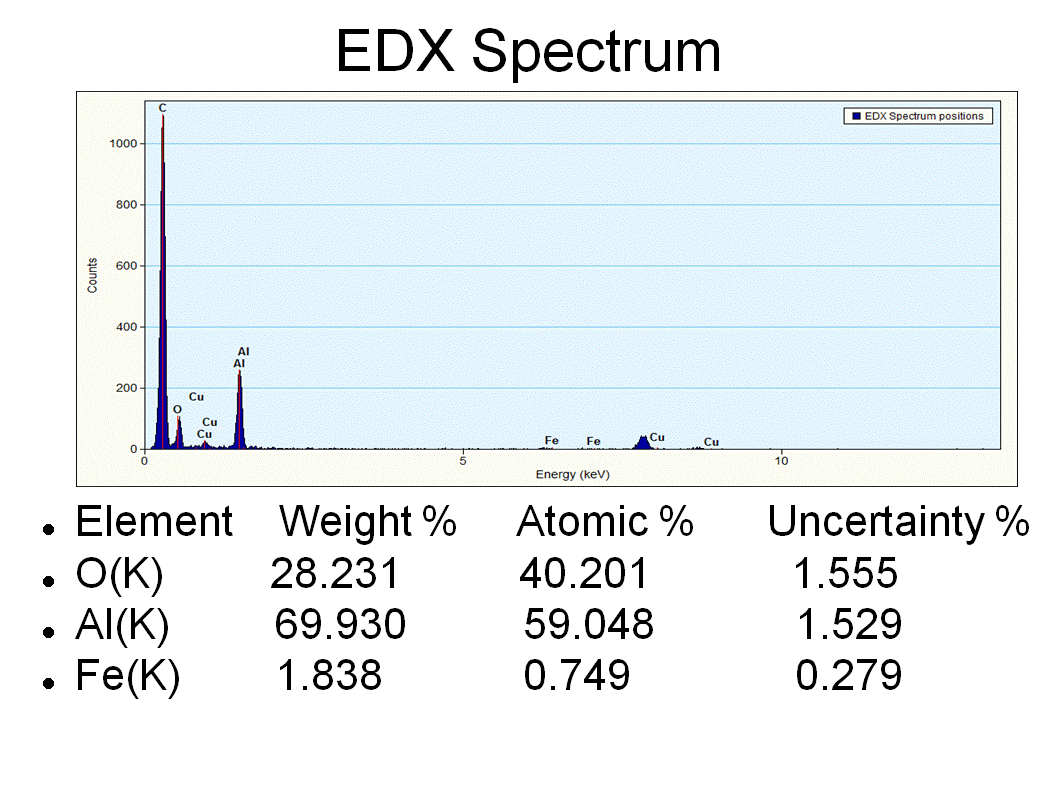
Figure S9: shows a) the HAADF-STEM, b) TEM-EDX of the un-purified ACFL550 catalyst.


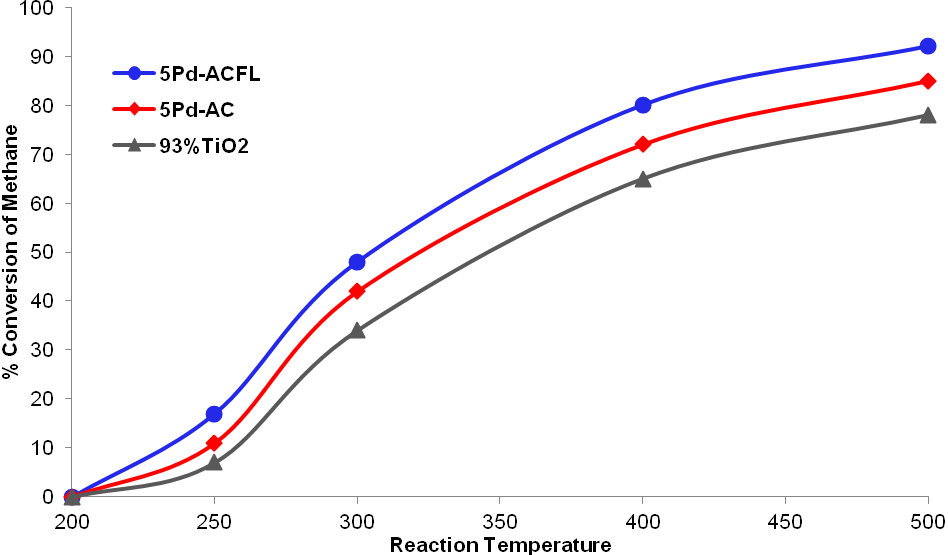


**Figure S10:** The catalytic conversion profiles of methane oxidation over 5Pd-ACFL (5 wt% Pd, 2 wt% Pt on 17.5% TiO_2_/ ACFL550,5Pd-AC (5 wt% Pd, 2 wt% Pt on 17.5% TiO_2_/ AC550 along with the mono support component, without acidic support, 93% TiO_2_(5 wt% Pd, 2 wt% Pt on 93% TiO_2_) under reaction temperatures of 200-500 °C with a GHSV of 100,000 mL g^-1^ h^-1^.

**a)**

**Table S1**: Lattice parameters and space group for the homemade aluminium chloride hexahydrate

| Lattice parameters and space group | This work | Ref.[1968] |
| --- | --- | --- |
| Crystal system | Trigonal | (hexag Trigonal onal) |
| Space group | R-3c | R-3c |
| a/Å | 11.8260(2) | 11.827 ± 0.006 |
| b/Å | 11.8260(2) | - |
| c/Å | 11.9064(3) | 11.895 ± 0.003 |
| α/° | 90 | 90 |
| β/° | 90 | 90 |
| γ/° | 120 | 120 |
| Volume/Å^3^ | 1442.07(5) | - |
| Z | 27 | - |
| Crystal density (ρ_calc_ g/cm^3^) | 1.667 | 1.644 |
| Temperature (K) | 291.58(10) | 295 |

**Table S2**: Lattice parameters and space group for the homemade aluminium nitrate nonahydrate

| Lattice parameters and space group | This work | Ref.[1983] |
| --- | --- | --- |
| Crystal system | Monoclinic | Monoclinic |
| Space group | P2_1_/c | P2_1_/c |
| a/Å | 13.85684(14) | 13.8937 (14) |
| b/Å | 9.45095(10) | 9.6258 (7) |
| c/Å | 10.87930(13) | 10.9127 (7) |
| α/° | 90 | 90 |
| β/° | 95.4618(9) | 95.66 (1) |
| γ/° | 90 | 90 |
| Volume/Å^3^ | 1418.29(3) | 1452.3 (2) |
| Z | 26 | - |
| Crystal density (ρ_calc_ g/cm^3^) | 1.766 | - |
| Temperature (K) | 123.9 (10) | 295 |

**Table S3:** ICP-OES data of the % in the single crystals produced from Al foil along with the γ-Al_2_O_3_ produced from commercial precursor and Al foil waste, where FL and Com are referred to the foil and commercials precursors, respectively.

| Phases | Compound name | % Al |
| --- | --- | --- |
| Single crystals | Al(NO_3_)_3_.9H_2_O^FL^ | 7.17 |
|  | Al(NO_3_)_3_.9H_2_O^Com^ | 7.09 |
|  | AlCl_3_.6H_2_O^FL^ | 11.13 |
|  | AlCl_3_.6H_2_O^Com^ | 11.17 |
| γ-Al_2_O_3_ | ACFL550 | 45.79 |
|  | AC550 | 45.21 |
